# An Approach of Manufacturing High-Molecular-Weight CNT-Filled Epoxy Composite

**DOI:** 10.3390/ma18020264

**Published:** 2025-01-09

**Authors:** Florence Acha, Talya Scheff, Nathalia Diaz Armas, Joey Mead, Stephen Johnston, Jinde Zhang

**Affiliations:** Department of Plastics Engineering, University of Massachusetts Lowell, Lowell, MA 01854, USA; florence_acha@student.uml.edu (F.A.); talya_scheff@student.uml.edu (T.S.); nathalia_diazarmas@student.uml.edu (N.D.A.); joey_mead@uml.edu (J.M.); stephen_johnston@uml.edu (S.J.)

**Keywords:** composites, CNT-filled epoxy, isothermal curing, ramped curing, curing dynamics, injection molding

## Abstract

Epoxy nanocomposites are widely used in various applications because of their excellent properties. Different types of manufacturing techniques are used to produce epoxy composites based on various fillers, molecular weight, and applications required. The physical properties and chemical structure of epoxy resin help in determining the method for its manufacturing. Coatings and adhesive formulations are prepared using high- molecular-weight epoxies, whereas epoxy nanocomposites require low-molecular-weight epoxies due to ease of manufacturing. A low-molecular-weight epoxy can provide high crosslink density to the epoxy but may also cause inherent brittleness in epoxy nanocomposites. Further, the addition of CNTs may also cause more brittleness in the final product. In this work, the authors have developed a method to process composites based on high-molecular-weight epoxy reinforced with high loading of CNTs (15 wt.%). The high molecular weight will bring lots of challenges during manufacturing. In this paper, a novel manufacturing technique based on separate molding and curing conditions to produce highly concentrated CNT-filled epoxy with high-molecular-weight epoxy resin is described, achieving excellent mechanical properties, good toughness, and high electrical conductivity in an efficient, low-cost, environmentally friendly, and high-volume way. The findings demonstrated improvements in these mechanical properties compared to conventional systems. They also highlight the potential of the novel method to develop advanced composite materials which can revolutionize industrial sectors such as aerospace, automotives, and electronics where structural integrity and thermal stability are important.

## 1. Introduction

Epoxy is well known as one of the most important thermosets because of its versatility in physical and mechanical properties (Kern et al., 1994; Bratychak et al., 1999) [1,2]. Different combinations of epoxy pre-polymer and curatives can be formulated for specific mechanical property requirements. Usually, a high crosslinking degree is needed in cured epoxy systems to achieve desired properties, such as high heat deflection (i.e., high glass transition temperature) along with high stiffness and strength, excellent resistance to chemical corrosion, fatigue, and so on (Bilyeu et al., 1999) [3]. The processing of epoxy is also simpler compared to other thermosets or thermoplastics because there are low shrinkage and the formation of almost no volatile by-products during the reaction. Due to the ease of application and excellent physical and mechanical properties, epoxies have been used in a wide range of applications, including coatings, protectants, adhesives, structural materials, electric encapsulates, and so on (Bilyeu et al., 1999) [3].

Although highly crosslinked epoxy possesses lots of desirable mechanical properties, the inherent brittleness restricts its applications in structural materials, aerospace, sports goods, and automobiles. Therefore, many researchers have focused on the improvement of fracture toughness in epoxy systems. In general, there are two common ways: the first method includes changing the structure of epoxy chemically to modify the epoxy backbone from a rigid structure to a more flexible structure or decreasing the crosslink density either by increasing the pre-polymer molecular weight or by reducing the functionality of curatives. This method is highly technical and costly. The other more common method to improve the fracture toughness is the incorporation of a dispersed tougher phase in an epoxy matrix, which could be rubber (Kou et al., 2018) [4] or thermoplastics (Velthem et al., 2016) [5]. Although this approach can toughen epoxy efficiently, the inclusion of a softer phase causes a decrease in other mechanical properties like stiffness.

Recently, incorporation of solid fillers in the epoxy matrix has been becoming more popular compared to rubber toughening systems. Although this method provides lesser toughness compared to rubber toughening systems, the improvements in stiffness and modulus properties are significant. Such reinforcing elements can be fibers including glass fiber (Saravanakumar et al., 2020) [6], carbon fiber (Feng et al., 2020) [7], aramid fiber (Nasser et al., 2019) [8], and boron fiber (Agrawal et al., 2020), [9], which are usually used commercially in industry. They can also be fillers with different kinds of geometry, such as spherical, layered, tubular, or crystal structures, etc., which include silica (Picu et al., 2019) [10], nanoclay (Chee et al., 2020) [11], graphite (Joshi et al., 2020) [12], cellulose (Nair et al., 2019) [13], and carbon nanotubes (Singh et al., 2019) [14]. Fiber-reinforced epoxies provide a better improvement in strength and fracture toughness compared to nanofiller-filled epoxies largely due to challenges in particle dispersion, tubes’ orientation, and interface bonding strength between the filler and the epoxy. However, recent studies have explored combining reinforcing fibers with nanoparticles in an epoxy matrix to address these limitations and further enhance performance, despite the low molecular weight of epoxy resin (Sergey et al., 2023) [15]. Also, the easy processability, relatively low cost, and versatility successfully counteract their limitations and make such nanocomposites have broad applications in those fields which require less demanding mechanical properties, such as consumer goods, interior decoration in airplanes and automobiles, electrical conductivity, thermal conductivity, antistatic, EMI shielding, and so on (Pillai et al., 2011) [16].

Carbon nanotubes (CNTs), formed by rolling single, double, or multiple layers of graphene onto themselves, have become very popular in composite-manufacturing industries due to their excellent mechanical properties (Young’s modulus > 1 TPa) (Lu, 1997) [17], flexibility (Despres et al., 1995) [18], tensile strength > 50 GPa (Iijima et al., 1996) [19], high thermal and electrical conductivity (Dai et al., 1996; Ebbesen et al., 1996) [20,21], etc. CNTs have been used in a variety of matrices, which include polypropylene (Kumar et al., 2002) [22], polycarbonate (Yoshino et al., 1999) [23], polystyrene (Qian et al., 2000) [24], poly (methyl methacrylate) (Jin et al., 2001) [25], poly (ethylene glycol) (Jin et al., 2000) [26], polyaniline (Cochet et al., 2001) [27], poly (vinyl alcohol) (Shaffer and Windle, 1999) [28], ceramics (Peigney et al., 2000) [29], and metals (Kuzumaki et al., 1998) [30]. A pronounced improvement of mechanical and electrical properties by the addition of CNTs has been reported (Sandler et al., 1999) [31].

CNT-filled epoxy composites are fabricated on a lab scale mainly by mechanical mixing (Sandler et al., 1999) [31], solution mixing (Zhu et al., 2003) [32], CNT array (Wardle et al., 2008) [33] buckypaper (Wang et al., 2003) [34], and yarn (Zhang et al., 2004) [35] impregnation methods. All these methods require low-molecular-weight epoxy resin and cannot produce highly concentrated CNT-filled epoxy. Resin transfer molding has been commercially used in industries to prepare CNT-filled epoxy composites (Cheng et al., 2009) [36]. This method can produce highly concentrated CNT-filled epoxy composites with super-highly aligned CNTs though it is also limited to the use of low-molecular-weight epoxy resin. Low-molecular-weight epoxy combined with a high concentration of CNTs cannot provide good toughness. Therefore, it would be interesting to study the properties of highly concentrated CNT composites based on high-molecular-weight epoxy resin. However, due to the high molecular weight, this kind of epoxy resin is mostly solid at room temperature. This has made it impossible or much harder to process such high-molecular-weight epoxy using existing processing strategies. A high-temperature processing technique is required. On the other hand, an elevated processing temperature could easily accelerate the cure rate and possibly make the scorch time too short for most traditional high-temperature processing techniques, for example, injection molding. In this case, several factors need to be considered and kept in balance, for instance, high-temperature hardener selection, injection molding processing control, epoxy cure kinetics, final properties of the cured samples, and the economics of the whole strategy. However, as per the authors’ knowledge, up to now, there has been very limited work reported in this area, even though such composites can provide excellent mechanical properties with higher toughness as well as good electrical and thermal conductivities.

In this study, a traditional injection molding technique was used to process highly concentrated CNT (15 wt.%) composites based on moderately high-molecular-weight solid epoxy resin. A specific high-temperature hardener, hexahydrophthalic anhydride (HHPA), was selected. Based on the curing behavior of the combination of the selected hardener and epoxy, injection molding parameters were optimized. After molding, post-curing was also studied, and the post-curing conditions were finally optimized. The final product had excellent mechanical properties as well as high electrical conductivity.

## 2. Materials and Methods

### 2.1. Materials

A CNT-filled epoxy masterbatch with 15 wt.% loading was purchased from Hyperion Catalysis International, Cambridge, MA. Based on the datasheet provided by the supplier, the CNTs are multiwalled with a 5 nm core (Potschke et al., 2002) [37]. The diameter range is stated to vary from 15 to 50 nm and the length is in the range of 1–10 micrometers. The base resin is EPON™ Resin 1007F (Houston, TX, USA) with 1700–2300 g/eq of weight per epoxide. The dispersion of CNTs is also characterized via transmission electron microscope (TEM–JEOL JEM-2100Plus TEM/STEM from JEOL Ltd., Tokyo, Japan) and scanning electron microscope (SEM–JOEL JSM 7401F from JEOL Ltd.) methods. The results are shown in Figures S1 and S2 in the Supplementary Materials. The results show that the dispersion of CNTs is very good even in the original masterbatch. After the injection molding process, the dispersion of CNTs becomes slightly better. The softening temperature of the masterbatch is 120–130 °C according to ASTM 3461. The hardener used to cure the masterbatch of CNT-filled epoxy was hexahydrophthalic anhydride (HHPA), manufactured by Polynt SpA in Scanzorosciate, Bergamo, Italy. The structural formula of the base resin (EPON™ Resin 1007F from Westlake Epoxy, a division of Westlake Corporation in Houston, TX, USA) is as follows: 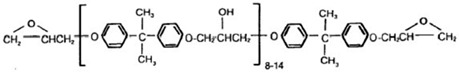


### 2.2. Molding Compound Preparation

The CNT-filled epoxy is in a pellet form and is solid below 120 °C. The selection of hardener plays a critical role in the fabrication of the compound as the processing temperature is above 120 °C. An appropriate hardener should have high heat resistance, long scorch time, and not be volatile at high temperatures. Based on these requirements, a solid hardener, hexahydrophthalic anhydride (HHPA), manufactured by Polynt SpA, was chosen. Before molding, CNT-filled epoxy masterbatch pellets and hardener were mixed at room temperature using a speed mixer (Hauschild Engineering DAC 150 FVZ, Hamm, Germany). The weight ratio of CNT-filled epoxy to HHPA was 100:3.276, which differs from the stoichiometric ratio.

### 2.3. Molding Method

For molding, a mixture of masterbatch and hardener was directly fed into injection molding (Sumitomo SE50EV-C110, Chiyoda City, Japan) to produce standard tensile and flexural bars. The optimized processing parameters are listed in Table 1, Table 2 and Table 3. During processing, the mold temperature was set at 46 °C and the cooling time was 20 s.

### 2.4. Curing

All the injection molded standard bars were cured in a regular oven. Both isothermal and ramped/stepped cure were carried out under different optimized temperatures and times. For the isothermal curing process, samples were cured in an oven at 120 °C for one week. For the ramped/stepped curing process in an oven, firstly, the temperature was ramped from room temperature to 120 °C at a rate of 5 °C/min. Then it stayed at 120 °C for 2 h to partially solidify the shape and then was ramped again to 200 °C at a rate of 2 °C/min and finally stayed at 200 °C for 14 h to obtain a gel structure. Then it was ramped from 200 °C to 250 °C at a rate of 1 °C/min to speed up the reaction and to obtain a higher cure degree. Finally, the oven was kept at 250 °C for an additional 12 h and then slowly cooled down to room temperature. After curing, all the samples were conditioned at 25 °C for at least 48 h before testing.

### 2.5. Characterization

#### 2.5.1. Mechanical Property Testing

Tensile properties were measured in the samples molded by injection molding using an Instron 6205 universal testing machine. The samples were molded into dumbbell-shaped specimens 162.61 mm in length, 12.80 mm in width, and 3.20 mm in thickness according to ASTM D638. Samples were stretched at a crosshead rate of 100 mm per minute with an extensometer attached to them. For flexural testing, standard rectangle bars molded by injection molding (3.23 × 12.89 × 96 mm) were used to perform three-point bending testing at a strain rate of 0.1 mm/min according to ASTM D790. Ten specimens for each composition were tested and their average was taken to ensure consistency.

#### 2.5.2. Thermal Property Testing

Thermal properties were studied by thermal gravimetric analysis (TGA) using TA Q50. Small pieces of about 20 mg were obtained by cutting molded samples and were then heated in air from room temperature to 800 °C at a constant rate of 20 °C/min.

#### 2.5.3. Rheological Property Testing

The cure behavior of epoxy was analyzed by a parallel plate rheometer (TA ARES G2, Lukens Drive New Castle, DE, USA). Samples were made by cutting molded bars into small disks with a 15 mm diameter. The molded bars were then tested on a parallel plate rheometer by ramping the temperature from 50 °C to 200 °C at a rate of 5 °C/min in oscillation mode. The frequency of oscillation was 1 Hz, and the strain amplitude was set 0.1%.

#### 2.5.4. Electrical Resistivity Testing

Electrical resistivity was measured in molded rectangle bars (flexural bars) in two different directions. One was parallel to the injection direction and the other was transverse to the injection direction. The measurement mode was the electric field direct current (DC). A two-point ohmmeter (DT0830B Digital Multimeter) was used for electrical resistivity testing. To ensure minimum resistance at the contact point of the electrode and specimen, conductive silver paste was painted on two ends of the tested specimen. The painting area was kept consistent.

## 3. Results and Discussion

### 3.1. Molding

**Figure 1:**
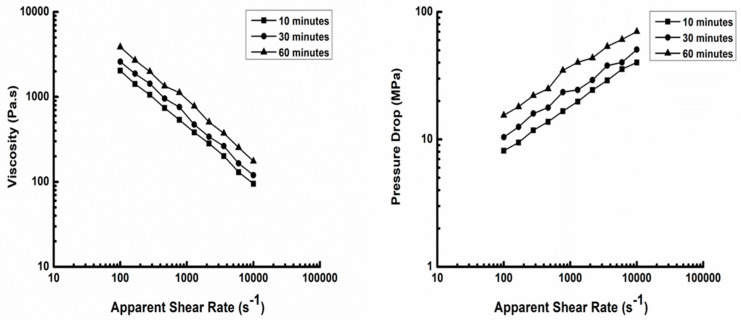
Capillary rheology testing on molding compound at 200 °C.

**Figure 2:**
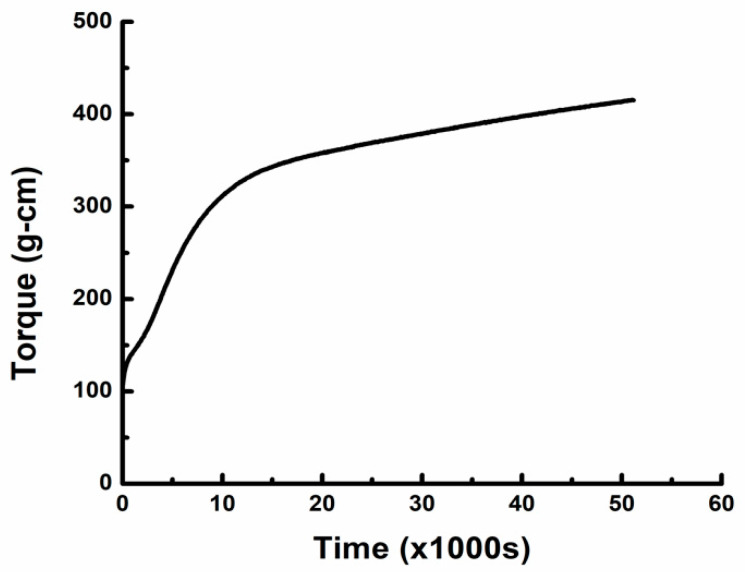
Parallel plate rheology testing on molding compound at 200 °C.

Traditionally, an epoxy molding compound is molded and cured either by casting in an open mold or compression molding. Both methods are suitable for only those molding compounds which have relatively low viscosity at a moderate temperature and a fast curing rate at a high temperature. Otherwise, either it will be difficult to fill the cavity or it may cause long cycle time. Also, these molding methods cannot make parts with complex shapes. The viscosity of our molding compound at a low shear rate ranging from 100–1000 s^−1^ (Figure 1) is high, about ten times higher than the viscosity at a high shear rate from 1000–10,000 s^−1^. Also, the curing analysis of our molding compound (Figure 2) demonstrates that, at 200 °C, curing occurred slowly in the first 60 min. Therefore, compression molding or casting was not feasible for our materials. Instead, injection molding would be a suitable technique based on our molding compound for the following reasons. First, the rheology behavior of our molding compound at a high shear rate (1000–10,000 s^−1^) (Figure 1) shows that the viscosity is low for an injection molding process and can be decreased further just by increasing the shear rate. Secondly, curing occurs to a very limited degree at 200 °C in 60 min, which ensures that there will be no scorch in the barrel during processing. Last, but not the least, our molding compound has a high softening temperature of 120 °C that means our materials can hold their shape on their own at a low temperature and there is no need to set the shape by curing under heat (thermoset) which is the usually performed for thermoset injection molding processing. This is extremely important because we can mold samples directly by a conventional injection molding machine using a cold mold. Further, we can also utilize the processing advantages of injection molding such as high-volume production, excellent consistency, good quality of parts, and of making complicated parts.

### 3.2. Isothermal Cure

**Figure 3:**
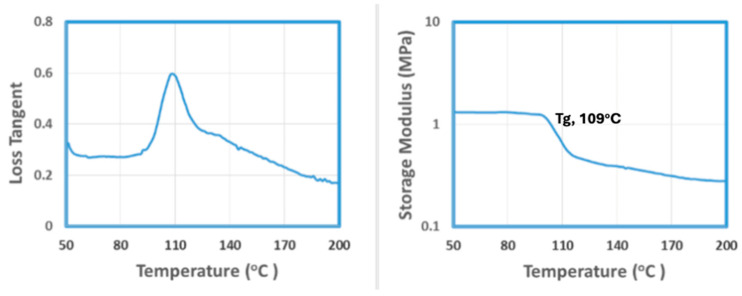
Tg test via parallel rheometer on CNT−filled epoxy without hardener.

Isothermal cure refers to curing of the sample at a constant temperature. Before curing, it is important to find the correct cure temperature boundary and then cure temperature. Theoretically, the low cure temperature limit is determined by the glass transition temperature of epoxy pre-polymer, and the upper limit should be the onset of degradation temperature of epoxy [37]. To obtain the glass transition temperature of virgin CNT-filled epoxy, the masterbatch of CNT-filled epoxy was also injection molded without hardener. The molded bars were then tested on a parallel plate rheometer at a ramping temperature from 50 °C to 200 °C at a rate of 5 °C/min in oscillation mode. The frequency of oscillation was 1 Hz, and the strain amplitude was set 0.1%. Results in Figure 3 indicate that the Tg of the masterbatch of CNT-filled epoxy was about 109 °C, which should be the bottom boundary of the cure temperature.

**Figure 4:**
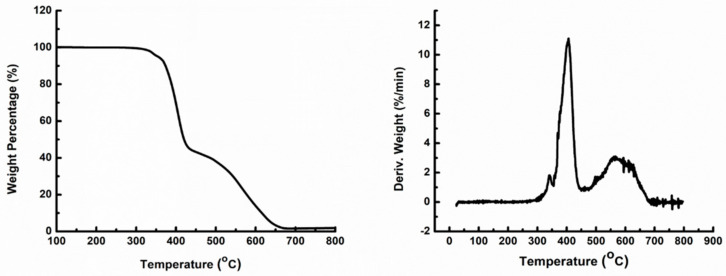
TGA test on CNT−filled epoxy without hardener.

For the upper boundary of the cure temperature, TGA was carried out on the same sample. Results (Figure 4) indicated that the onset of degradation temperature for the masterbatch of CNT−filled epoxy was about 320 °C, which should be the upper boundary of the cure temperature.

Based on the above analysis, three different temperatures were chosen to cure our injection molded bars. These were 200 °C, 250 °C, and 300 °C. A parallel rheometer was used to monitor the whole curing process under each different temperature to further understand the thermodynamic and kinetic behavior of curing. Activation energy (E_a_) is significant for understanding the thermodynamic behavior of any chemical reaction. It can be considered as the height of the potential barrier (sometimes called the energy barrier) separating two minima of potential energy (of the reactants and products of a reaction). For a chemical reaction to proceed at a reasonable rate, there should exist many molecules with energy equal to or greater than the activation energy. At a more advanced level, the Arrhenius Activation energy term from the Arrhenius equation (Equation (1)) is best regarded as an experimentally determined parameter that indicates the sensitivity of the reaction rate to temperature (Sun, 2002) [38].

(1) $$K=Ae^{\frac{-E_{a}}{RT}}$$

where *A* is the frequency factor for the reaction, *R* is the universal gas constant, *T* is the temperature (in kelvin), and *K* is the reaction rate coefficient. Even without knowing *A*, Ea can still be evaluated from the variation in reaction rate coefficients as a function of temperature (within the validity of the Arrhenius equation).

According to the literature (Sun, 2002) [38], for an epoxy curing reaction, the reaction rate coefficient can be demonstrated by the gel time. A high cure temperature is supposed to cause a shorter time to reach the gel point. Then the Arrhenius equation can be adapted to Equation (2), which correlates the gel time with cure temperature. Therefore, the activation energy can be obtained by measuring the different gel times under different cure temperatures.

(2) $$Lnt_{\mathrm{gel}}=c+\frac{Ea}{R}\cdot \frac{1}{T}$$

**Figure 5:**
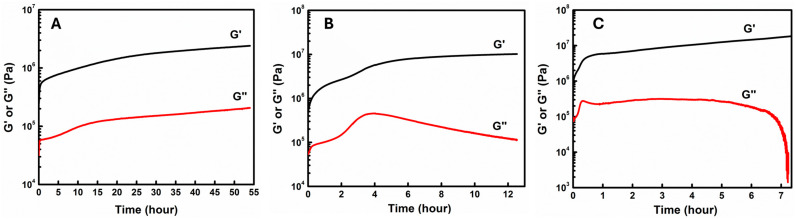
Dependence of Storage Modulus G′ and Loss Modulus G″ on Time at 200 °C (**A**), 250 °C (**B**), and 300 °C (**C**).

In this study, three different temperatures, 200 °C, 250 °C, and 300 °C, were used for isothermal curing of CNT-filled epoxy. Storage modulus (G′), loss modulus (G″), loss tangent (tanα), and complex viscosity (η*) were measured as a function of time to record the curing process. Figure 5 shows the typical variations of storage modulus G′ and loss modulus G″ with the time at different temperatures. During the entire curing process, the storage modulus G′ is greater than loss modulus G″, which indicates that our materials are more solid due to their high molecular weight and CNT content. This is different from ordinary epoxy systems, which usually show a more liquid-like (G′ < G″) character at the beginning of curing (Sun, 2002) [38]. In our system, curing occurred very fast in the initial period and then slowed down gradually as time and cure degree advanced. This is reasonable because, at the beginning of the cure, the mobility of curatives was high, which caused a high cure rate. However, as the cure degree increased, the viscosity of the whole cure system became higher. The mobility of curatives became much more constrained and the cure reaction rate was dominated by the diffusion of curatives. Thus, the net curing rate slowed down. Storage modulus increased with the advancement of the cure process and finally reached a plateau. In contrast, loss modulus increased due to the increment in molecular weight through the curing reaction (polymerization), reached a maximum, and then decreased due to the solidification of the epoxy system when it approached the gelled glass region. Therefore, the gel time should be earlier than the point when G″ reached the maximum.

**Figure 6:**
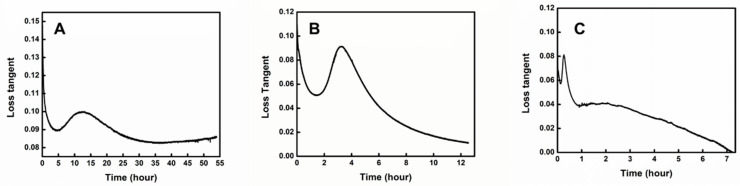
Dependence of loss tangent on Time at 200 °C (**A**), 250 °C (**B**), and 300 °C (**C**) measured at angular frequency of 1 Hz.

In this work, gel time was taken as the point when the loss tangent reached the maximum (peak). The loss tangent can indicate and quantify the material’s character, such as more liquid-like or solid-like. Before the gel point, the molecular weight increased with curing which increased G″, while G′ was also increased with curing due to a higher cure degree. Therefore, the loss tangent showed a wave-like shape and, after the gel point, the network started to form. Solidification of the epoxy system happened dramatically. G′ kept rising while G″ increased more and more slowly and eventually stopped growing and began to drop. As the loss tangent kept decreasing gradually, the peak of the loss tangent was reasonably assumed to indicate the gel point. Figure 6 and Table 4 show that the gel time was apparently affected by temperature. A higher temperature decreased gel time very significantly. According to Equation (2), the activation energy *E_a_* was finally obtained, which was 87.74 kJ/mol.

**Figure 7:**
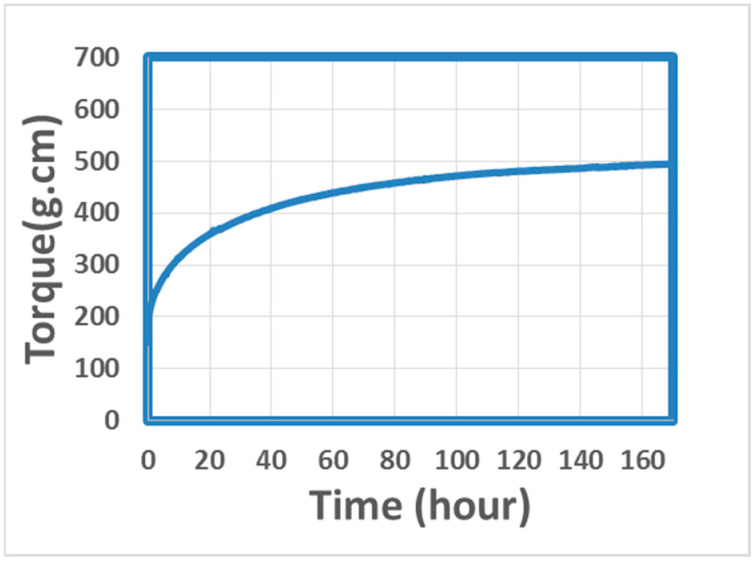
Cure process at 120 °C for one week.

Samples cured at three different temperatures were checked and measured. Results are shown in Table 5, which indicates that all the samples had defects. Bubbles were found on all the samples’ surfaces, while warpage happened for the samples cured at 300 °C. It is believed that bubbles occurred due to the degradation of epoxy under long-term heating. Warpage happened because the curing speed was too fast at 300 °C, which caused non-uniform temperature and cure degree distribution across the sample. Both of them caused non-uniform shrinkage across samples and finally led to warpage. Therefore, a lower cure temperature will be helpful to prevent such defects. So, samples were then cured at 120 °C for one week. The curing process was also measured by a parallel plate rheometer and the result is shown in Figure 7.

Samples cured at 120 °C showed excellent quality without any bubbles or warpage. Mechanical and thermal properties were tested to quantify the properties of cured parts. Table 6 shows some important properties related to flexural deformation. It indicates that curing improves samples’ flexural properties in all cases except for modulus. For example, the flexural strength was enhanced by about 130% from 34.6 MPa to 81.8 MPa. Almost 150% enhanced strain at break represented the ductility of samples. Moreover, the strain energy at break showing material toughness was increased by 500%. For tensile properties, results shown in Table 7 indicate similar trends to flexural properties.

**Figure 8:**
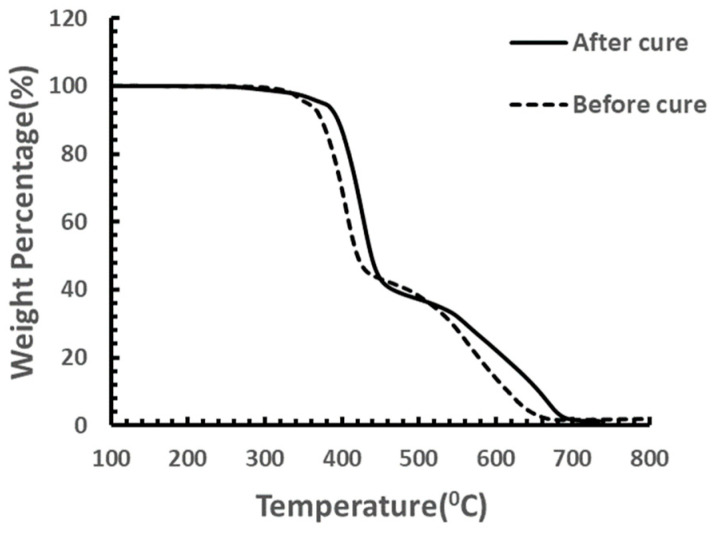
TGA testing on samples cured at 120 °C.

Besides mechanical properties enhancement, the thermal properties were also improved by curing. As shown in Figure 8, after curing, the parts’ maximum degradation temperature in air was enhanced by almost 20 °C.

Finally, the electrical resistivity of cured samples was also measured by a two-point ohmmeter. Results are listed in Table 8. First, in both directions, the electrical resistivity of samples was low (<22 Ω.cm). Additionally, the electrical resistivity of injection molded samples reflects anisotropy. Samples have much lower resistivity (10.4 Ω.cm) in the direction parallel to the injection direction (melt flow direction) than in the direction transverse to the injection direction (21.7 Ω.cm). This may be due to CNTs’ orientation in the melt flow direction by high shearing during injection. To confirm this point, TEM images need to be taken in future. The degree of such anisotropy can be controlled by changing injection molding parameters, like injection speed and mold temperature.

### 3.3. Ramped/Stepped Cure

As per the above discussion, only a low cure temperature (120 °C) was proper for curing samples in isothermal mode. A higher temperature caused defects on parts like bubbles and warpage. However, curing at 120 °C took almost one week, which will not be accepted in the industry. The proper method to find a balance between the cured parts’ quality and cure time (cost) may be stepped cure or staged cure. During stepped cure, samples are cured at different temperatures during various stages. In the early stages, samples are cured at a relatively low temperature to control the shrinkage and residual stress until the gel structure is formed. Then they are cured at an elevated temperature to speed up the cure rate and increase the final cure degree. This method can produce cured parts with excellent mechanical properties in a reasonable time. Shrinkage, warpage, residual stress, and degradation of epoxy can also be kept as low as possible.

**Figure 9:**
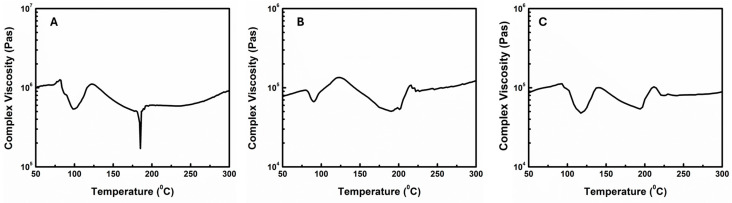
Dynamic scanning cure of CNT-filled epoxy at 5 °C/min (**A**), 10 °C/min (**B**), and 20 °C/min (**C**).

**Figure 10:**
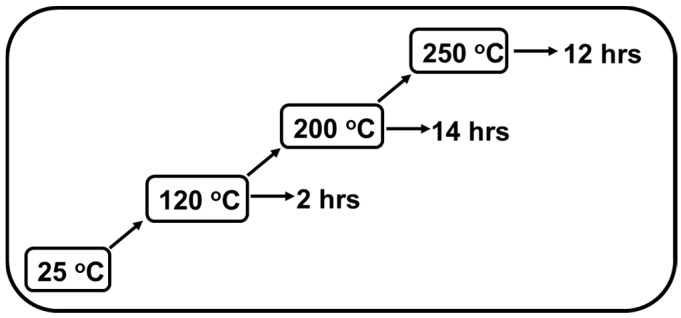
Ramped/stepped cure procedure.

Before starting the stepped cure, it is necessary to figure out the temperature and time for each stage. In this study, we used dynamic cure analysis on the epoxy system via a parallel plate rheometer to determine the temperatures. Samples were cured in dynamic mode at different temperature ramping rates (Figure 9). The complex viscosity was determined by temperature and cure degree. A higher temperature gives a lower viscosity, while a high degree of cure will increase viscosity due to increasing molecular weight. Therefore, the relationship between complex viscosity and temperature in a dynamic cure can be very complicated, which was demonstrated by the irregular shape of curves shown in Figure 9. In the temperature region of 50–80 °C, complex viscosity of the epoxy system slightly increased with temperature. This is because this region is a glass region of epoxy. Therefore, the viscosity was not affected by temperature, while curing also happened slowly. When temperature was ramped close to the Tg of epoxy (about 100 °C), the viscosity of the epoxy system dropped dramatically, while cure still happened slowly. However, at a temperature higher than the Tg, cure reaction speed was accelerated due to enhanced mobility of epoxy chains, which caused a rise in viscosity again. The viscosity just kept increasing until the softening point of epoxy (120–130 °C) was reached. After this point, the temperature had more influence on viscosity. Even though the cure reaction rate kept increasing with temperature, the absolute value was still not big enough to offset the effect of temperature on viscosity. Therefore, there was a long gradual dropping period for viscosity after the epoxy started to soften. This period stopped at about 185–200 °C and, after that, viscosity kept increasing with temperature. This indicated that the curing rate after 200 °C was apparently high and had a dominant influence on the melt viscosity of our cure system. Based on the above discussion, we designed the final ramped/stepped cure procedure which is shown in Figure 10. Firstly, the oven temperature was ramped from room temperature to 120 °C at a rate of 5 °C/min. Then it stayed at 120 °C for 2 h to partially solidify the shape and then was ramped again to 200 °C at a rate of 2 °C/min and finally stayed at 200 °C for 14 h to obtain a gel structure. Then it was ramped from 200 °C to 250 °C at a rate of 1 °C/min to speed up the reaction and to obtain a higher cure degree. Finally, the oven was kept at 250 °C for an additional 12 h and then slowly cooled down to room temperature. The final degree of cure can be controlled by changing the curing time at 250 °C.

Samples after ramped/stepped curing had no defects like bubbles or warpage. All the samples were then characterized in terms of their mechanical, thermal, and electrical properties. Flexural and tensile properties are listed in Table 9 and Table 10, which indicate that curing improved samples’ mechanical properties in all the cases. For example, the flexural strength was enhanced by about 98% from 34.6 MPa to 68.6 MPa. Strain at break representing the ductility of samples was also enhanced by almost 100%. Moreover, the strain energy at break representing the material toughness was enhanced by 418%. For tensile properties, results are shown in Table 10, indicating similar trends to flexural properties.

**Figure 11:**
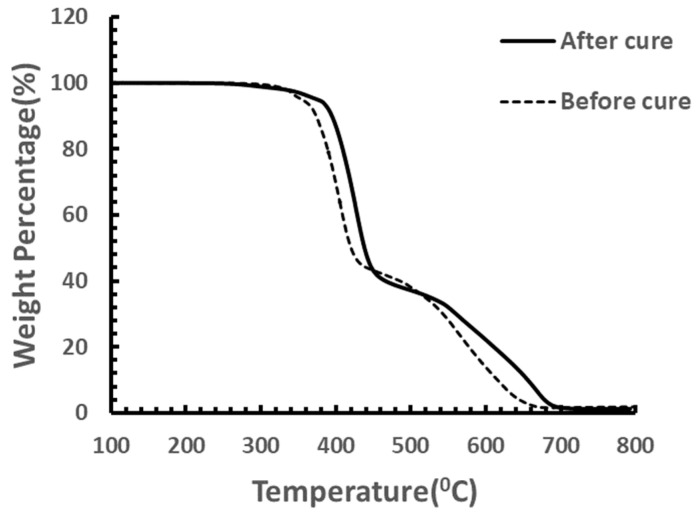
TGA testing on samples after stepped cure.

Besides mechanical properties enhancement, the thermal properties were also improved by curing. As shown in Figure 11, after curing, the parts’ onset of degradation temperature in air was enhanced by almost 50 °C while the maximum degradation temperature remained constant.

Finally, the electrical resistivity of cured samples was also measured by a two-point ohmmeter (Table 11). Compared to the samples cured at 120 °C, the electrical resistivity slightly decreased for samples cured by stepped curing. Additionally, anisotropy of conductivity was still observed and had a similar ratio to samples cured at 120 °C.

## 4. Conclusions

In this study, a manufacturing technique was developed specifically for composites based on high-molecular-weight epoxy reinforced with a very high loading of CNTs (15 wt.%). Unlike traditional manufacturing methods for epoxy composite, in which molding and curing are combined in one process, our method was based on separate molding and curing conditions. Traditional injection molding with a cold mold was used to obtain perfect parts with the desired shape. Then curing of these molded samples was initiated and finished in the oven. This design can make molding and curing work separate and independent, which can dramatically increase the production rate without sacrificing parts’ quality. Intensive study on both injection molding processing and curing conditions was carried out. The processing parameters for injection molding were also optimized. Also, to obtain an in-depth understanding of the epoxy curing behavior, rheological properties of the cure system were studied in different conditions by a parallel plate rheometer. Both thermodynamic and kinetic behaviors were investigated, which eventually helped to find out the appropriate cure conditions for our samples. Results from mechanical, thermal, and electrical properties testing showed that our technique was able to make parts with excellent mechanical properties like high strength, toughness, and thermal stability as well as high and anisotropic electrical conductivity. Our method was proven to be an efficient, low-cost, high-volume production, and environmentally friendly way to manufacture composites based on high-molecular-weight epoxy reinforced with a very high loading of CNTs (15 wt.%).

The study successfully demonstrated an innovative approach to manufacturing high-molecular-weight CNT-filled epoxy composites, which yielded materials with superior mechanical, thermal, and electrical properties. Key takeaways from the study include the optimized processing methodology in controlling dispersion and curing processes, the superior performance metrics seen in the improved thermal stability, electrical conductivity, and mechanical strength compared to conventional epoxy systems, and the industrial potential to satisfy the demand for durable materials in aerospace, automotive, and electronic applications.

## 5. Challenges and Future Prospects of Work

The primary challenges associated with this work include achieving improved interfacial adhesion between the CNT fibers and the epoxy matrix, as well as attaining a uniform dispersion of CNTs within the matrix. Scalability may also pose a significant hurdle, especially for industrial applications. Furthermore, precise control of curing conditions and alignment of CNT fibers could introduce additional processing complexities.

Despite these challenges, the work offers promising future prospects. Advanced functionalization techniques can be explored to improve the compatibility of CNTs with the epoxy matrix, thereby enhancing interfacial properties. The integration of hybrid fillers, combining CNTs with other materials, presents an opportunity to develop multifunctional composites. Additionally, automation and process optimization hold great potential for ensuring uniformity and maintaining the quality of composites during large-scale production.

This research also highlights opportunities for expanded applications in emerging fields, such as renewable energy systems and high-performance structural components. Furthermore, the incorporation of sustainable practices, including the use of bio-based materials, aligns with global environmental goals, making this work a critical step towards the development of sustainable advanced composites.

## Figures and Tables

**Table 1:** Barrel temperature setting parameters (°C).

Z6	Z5	Z4	Z3	Z2	Z1	Z0	Hopper
200	190	185	175	170	160	165	23

**Table 2:** Injection setting parameters for screw.

	Shot Size	1 st	V-P
Screw position (mm)	50	6	5
Injection speed (mm/s)	0	150	100

**Table 3:** Injection setting parameters for pressure.

Pressure (MPa)	Time (s)
65	3

**Table 4:** Gel Time at Different Temperatures and the Activation Energy.

Temperature (°C)	200	250	300
t_gel_ (s)	49,469	11,893	964
*E_a_* (KJ/mol)	87.74		

**Table 5:** Sample quality after isothermal cure.

Temperature (°C)	Time (Hour)	Sample Quality
200	89	A few bubbles
250	13	Fair amount of bubbles
300	10	Lots of bubbles and warpage

**Table 6:** Flexural testing of samples cured at 120 °C.

	Modulus (MPa)	Stress at Break (MPa)	Strain at Break (%)	Strain Energy at Break (J/m^2^)
Before cure	3961 (100)	34.6 (3.9)	0.9 (0.1)	0.035 (0.008)
After cure	3885 (30)	81.8 (6.7)	2.3 (0.2)	0.21 (0.03)
Enhancement (%)	−2	136	155	500

Note: values in parentheses are standard deviations.

**Table 7:** Tensile testing of samples cured at 120 °C.

	Modulus (MPa)	Stress at Break (MPa)	Strain at Break (%)	Strain Energy at Break (J/m^2^)
Before cure	3200 (58)	15.5 (3)	2.2 (0.3)	0.6 (0.15)
After cure	3900 (40)	42.6 (5)	4.4 (0.3)	2.7 (0.68)
Enhancement (%)	22	175	100	350

Note: values in parentheses are standard deviations.

**Table 8:** Electrical resistivity test on CNT-filled epoxy cured at 120 °C.

Direction	Resistivity (Ω.cm)
Parallel to Flow	10.4
Transverse to Flow	21.7

**Table 9:** Flexural testing on samples after stepped cure.

	Modulus (MPa)	Stress at Break (MPa)	Strain at Break (%)	Strain Energy at Break (J)
Before cure	3961 (100)	34.6 (3.9)	0.9 (0.1)	0.035 (0.008)
After cure	4188 (45)	68.6 (4.2)	1.8 (0.1)	1.5 (0.02)
Enhancement (%)	6	98	100	418

Note: values in parentheses are standard deviations.

**Table 10:** Tensile testing on samples after stepped cure.

	Modulus (MPa)	Stress at Break (MPa)	Strain at Break (%)	Strain Energy at Break (J)
Before cure	3200 (58)	15.5 (3)	2.2 (0.3)	0.6 (0.15)
After cure	4137 (50)	35.8 (4)	3.7 (0.2)	1.9 (0.3)
Enhancement (%)	30	130	68	216

Note: values in parentheses are standard deviations.

**Table 11:** Electrical resistivity test on CNT-filled epoxy after stepped cure.

Direction	Resistivity (Ω.cm)
Parallel to Flow	10.0
Transverse to Flow	20.9

## Data Availability

The original data presented in the study are openly available in https://figshare.com/s/9ecdbf589cafb8d84f2d (accessed on 21 November 2024) at DOI:10.6084/m9.figshare.28121858.

## Supplementary Materials

The following supporting information can be downloaded at: https://www.mdpi.com/article/10.3390/ma18020264/s1, Figure S1: TEM images of CNTs dispersion in masterbatch before and after injection molding process; Figure S2: SEM images of CNTs dispersion in masterbatch before and after injection molding process; Figure S3: Images of samples for tensile and flexure test.
