# Supplementary material for: An Approach of Manufacturing High-Molecular-Weight CNT-Filled Epoxy Composite
Source: Materials (Basel). 2025 Jan 9;18(2):264. doi: 10.3390/ma18020264 (PMC11767119; doi:10.3390/ma18020264)
Supplement: Supplementary file 1 [file materials-18-00264-s001.zip › materials-3359271-supplementary.pdf]

Supplementary Materials

Morphological characterization

Transmission electron microscopy (TEM) was used to obtain images on microtomed samples (~200 nm thick) using a Philips EM400T at an accelerating voltage of 100 kV to study the dispersion of CNTs in Epoxy/CNTs masterbatch.

Scanning electron microscopy (SEM) images were taken on a field-emission scanning electron microscope (JSM 7401F, JEOL Inc.) typically at an electron energy of 2 to 10 kV. Coated samples were sputtered with several nanometers of a thin gold film to enhance the conductivity and avoid severe charging during high magnification scanning.

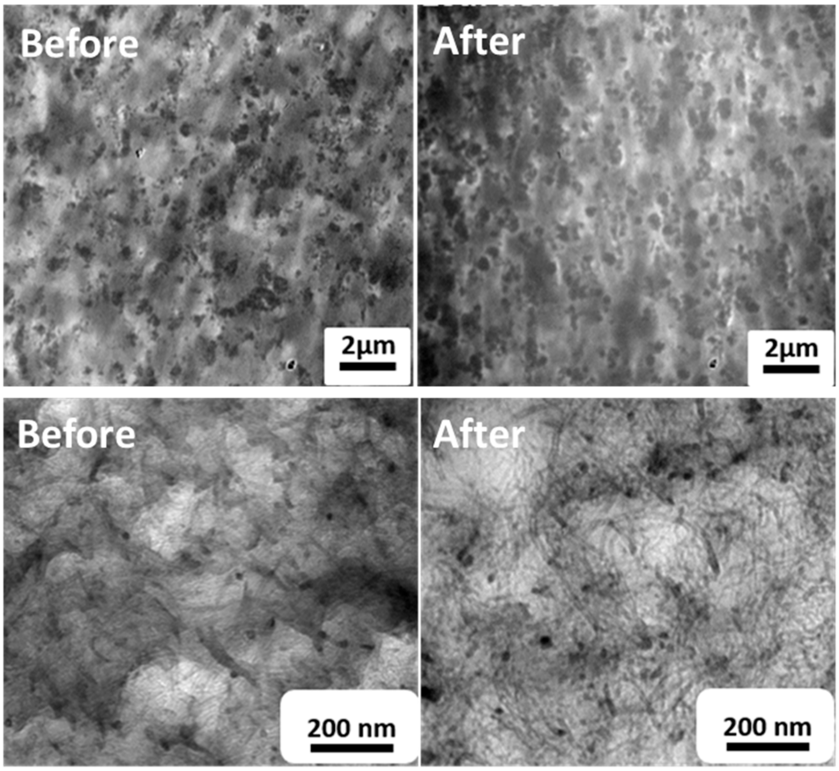

Figure S1. TEM images of CNTs dispersion in masterbatch before and after injection molding process.

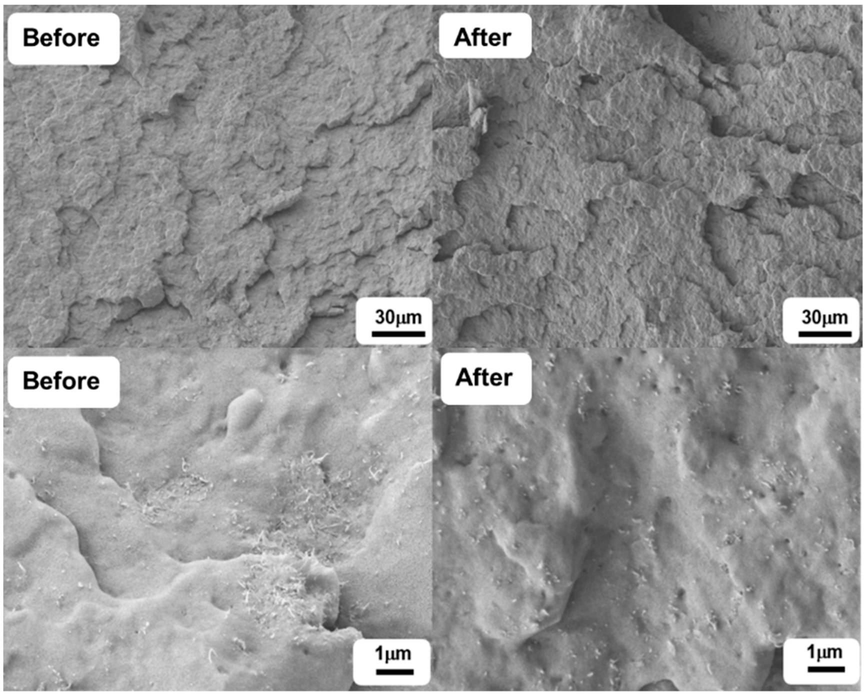

Figure S2. SEM images of CNTs dispersion in masterbatch before and after injection molding process.

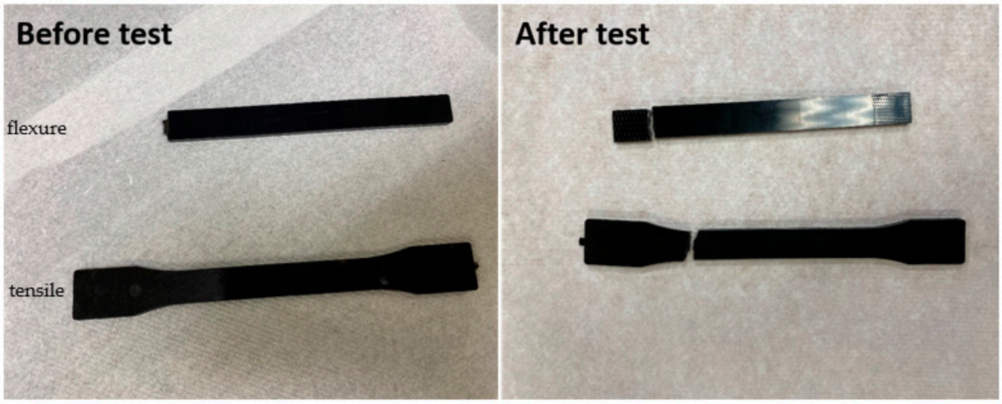

Figure S3. Images of samples for tensile and flexure test.
